# Draft Genome Sequences of Mycoplasma mycoides subsp. *mycoides* Strains APF9 and AP108, Isolated in Nigeria in 2014 to 2016

**DOI:** 10.1128/MRA.00783-19

**Published:** 2019-09-19

**Authors:** M. Di Federico, M. Orsini, M. Ancora, M. Marcacci, M. Di Domenico, I. Krasteva, K. Zilli, J. A. Musa, M. I. Francis, F. Sacchini, M. Scacchia, C. Cammà

**Affiliations:** a Faculty of Biosciences and Agro-Food and Environmental Technology, University of Teramo, Teramo, Italy; b Istituto Zooprofilattico Sperimentale dell′ Abruzzo e del Molise G. Caporale (IZSAM), Teramo, Italy; c Faculty of Veterinary Medicine, University of Maiduguri, Maiduguri, Nigeria; d Faculty of Veterinary Medicine, Ahmadu Bello University, Zaria, Nigeria; University of Maryland School of Medicine

## Abstract

Mycoplasma mycoides subsp. *mycoides* is the etiological agent of contagious bovine pleuropneumonia (CBPP). While several findings on CBPP prevalence in Nigeria were documented, no data were reported about the genomic characterization of Nigerian M. mycoides subsp. *mycoides* strains. Here, we present the draft genome sequences of two novel M. mycoides subsp. *mycoides* strains isolated in Nigeria.

## ANNOUNCEMENT

Mycoplasma mycoides subsp. *mycoides* is the causative agent of contagious bovine pleuropneumonia (CBPP), a severe contagious respiratory disease affecting cattle, which is included among the Office International des Epizooties (OIE) diseases (1).

Most countries have eradicated CBPP (http://www.oie.int/en/animal-health-in-the-world/official-disease-status/cbpp), but the disease still remains endemic in sub-Saharan Africa, including Nigeria, where it causes, directly or indirectly, economic losses of about $2 billion annually (2).

Epidemiological studies were performed in the northeastern region of Nigeria (3, 4), but no extensive work was carried out on the genomic characterization of Nigerian M. mycoides subsp. *mycoides* isolates, which would be of great importance in order to clarify the relationship among strains circulating in different regions of the country, providing support to CBPP outbreak investigations and disease control.

The draft genome sequences of two novel M. mycoides subsp. *mycoides* strains, named APF9 and AP108, are presented here. The first strain, APF9, was isolated in 2014 from pleural fluid of a cow showing pathological lesions referring to CBPP at slaughter in the Yola Modern Abattoir (Adamawa State, Nigeria) (5). The AP108 strain was isolated in 2016 from pleural fluid of a regularly slaughtered cow in the Yola Modern Abattoir.

The isolates were grown at 37°C under 5% CO_2_ in pleuropneumonia-like organism (PPLO) broth and agar (Difco) supplemented as previously described (6). The genomic DNA was extracted using a Maxwell 16-cell DNA purification kit (Promega), and a PCR-based test (7) was used for the identification of M. mycoides subsp. *mycoides*. Library preparation was carried out using a Nextera XT library prep kit (Illumina, Inc.), according to the manufacturer’s manual. The libraries were loaded onto an Illumina 300-cycle NextSeq 500/550 mid output reagent cartridge 2 kit and sequenced on an Illumina NextSeq 500 platform, producing 150-bp paired-end reads.

A total of 1,782,894 and 1,146,316 reads were obtained for the APF9 and AP108 strains, respectively, corresponding to theoretical coverages of 450× and 285×, respectively (average read length, 150 bp). After trimming performed using Trimmomatic 0.35 (8) (ILLUMINACLIP:NexteraPE-PE:2:30:10, LEADING:25, TRAILING:25, SLIDINGWINDOW:20:25, MINLEN:36), *de novo* assembly was carried out using SPAdes 3.11 (9), with default parameters for Illumina reads. Contigs longer than 200 bp were ordered by ABACAS 1.3 (10) using the genome of M. mycoides subsp. *mycoides* strain T1/44 (GenBank accession number CP014346) as a reference. Gaps were then filled using Pilon 1.23 (11) and GapFiller 2.1.1 (12).

The final assemblies, consisting of 3 contigs for the APF9 strain (total length, 1,203,001 bp; GC content, 23.96%; contig lengths, 999,440, 124,562, and 78,999 nucleotides) and 2 contigs for the AP108 strain (total length, 1,203,800 bp; GC content, 23.95%; contig lengths, 1,124,870 and 78,930 nucleotides), were submitted to GenBank, and annotation using the Prokaryotic Genome Annotation Pipeline (PGAP) pipeline (13) was requested. Genome annotation returned 1,159 genes for both the APF9 (including 936 protein-coding genes and 184 pseudogenes) and AP108 (including 935 protein-coding genes and 185 pseudogenes) strains. Two rRNA operons were annotated for each strain, and 30 tRNAs and 3 noncoding RNAs (ncRNAs) were identified. No plasmids were found in the APF9 and AP108 strains.

### Data availability.

The genome sequences were deposited in GenBank under the following accession numbers: VCPG00000000 (strain APF9) and VCPH00000000 (strain AP108). The Illumina raw reads were deposited in the NCBI Sequence Read Archive (SRA) under the accession numbers SAMN11775870 (strain APF9) and SAMN11775871 (strain AP108).
